# Synergistic Reinforcement of Si_3_N_4_ Based Ceramics Fabricated via Multiphase Strengthening under Low Temperature and Short Holding Time

**DOI:** 10.3390/ma16186163

**Published:** 2023-09-11

**Authors:** Jun-Wei Huang, Xiao-An Lv, Xiao-Feng Dong, Chang-Chun Ge

**Affiliations:** Institute of Powder Metallurgy and Advanced Ceramics (IPMAC), School of Materials Science and Engineering, University of Science and Technology Beijing (USTB), Beijing 100049, China

**Keywords:** silicon nitride, low temperature, microstructure, mechanical properties, synergistic reinforcement

## Abstract

Si_3_N_4_ ceramic as a tool material shows promising application prospects in high-speed machining fields; however, the required high mechanical properties and low-cost preparation of Si_3_N_4_ ceramic tool materials restrict its application. Herein, synergistic reinforced Si_3_N_4_ ceramic tool materials were fabricated by adding β-Si_3_N_4_ seeds, inexpensive Si_3_N_4_ whiskers and TiC particles into coarse commercial Si_3_N_4_ powder (D_50_ = 1.5 μm), then sintering by hot-pressing with low temperature and short holding time (1600 °C—30 min—40 MPa). The phase assemblage, microstructure evolution and toughening mechanisms were investigated. The results reveal that the sintered Si_3_N_4_ ceramics with synergistic reinforcement, compared to those with individual reinforcement, present an enhancement in relative density (from 94.92% to 97.15%), flexural strength (from 467.56 ± 36.48 to 809.10 ± 45.59 MPa), and fracture toughness (from 8.38 ± 0.19 to 10.67 ± 0.16 MPa·m^1/2^), as well as a fine Vickers hardness of 16.86 ± 0.19 GPa. Additionally, the various reinforcement modes of Si_3_N_4_ ceramics including intergranular fracture, crack deflection, crack bridging and whiskers extraction were observed in crack propagation, arising from the contributions of the added β-Si_3_N_4_ seeds, Si_3_N_4_ whiskers and TiC particles. This work is expected to serve as a reference for the production of ceramic cutting tools.

## 1. Introduction

Silicon nitride (Si_3_N_4_) ceramics as engineering structural ceramics can be applied in cutting tool materials owing to their superior overall performances [1,2], including excellent mechanical properties [3,4,5], low thermal expansion, self-lubrication, high temperature resistance [6], and chemical stability [7], etc. Compared with other ceramic cutting tools [8,9], Si_3_N_4_ ceramics as cutting tool materials are more suitable for applications in the field of metal cutting [10,11], which will reduce manufacturing costs and enhance machining efficiency in actual production. In particular, Si_3_N_4_ ceramic cutting tools [12] with high hardness can be also applied in machining difficult-to-machine materials [13] such as cemented carbide, nickel-base superalloy [14], grey cast iron, etc. Unfortunately, the application extents of Si_3_N_4_ ceramic cutting tools [15] in high-speed machining are generally restricted by expensive raw materials, inferior comprehensive mechanical properties, high-cost preparation with high sintering temperature (≥1700 °C) [16,17] and long holding time (≥1 h) [18].

Moreover, ceramics-based cutting tools occupy a small volume in cutting tools for metal machining, about 3%. The base problem is that the low fracture toughness and high brittleness of ceramics [19] seriously restrict the development of Si_3_N_4_ ceramic cutting tools. For this reason, increased toughness and strength are a good solution to the problem of increased usage of ceramics in this industrial sector. Although many studies have focused on the performance of Si_3_N_4_ ceramic materials, there are still some shortcomings. Currently, to enhance the strength and toughness of Si_3_N_4_ ceramics, various approaches [9,20] of reinforcement have been carried out by employing particles reinforcement [21], whiskers reinforcement [22], self reinforcement [23], gradient structure and synergistic reinforcement [24] etc. Self-reinforced Si_3_N_4_ ceramics are common and achieved via tailoring the α/β-Si_3_N_4_ phases ratio under sintering conditions of high temperature and long holding time [1,10,23]. The synergistic reinforcements method is accomplished by coordinating multiple strengthen phases, which significantly surpass the single strengthen phase [25,26]. Zou et al. [27] revealed that the addition of nanoscale TiN particles and Si_3_N_4_ whiskers contribute to the promotion of microstructural evolution and mechanical properties of Si_3_N_4_-based ceramic tool materials. Another work [28] about the preparation of laminated Si_3_N_4_/SiCw ceramics was also conducted under hot-pressed sintering conditions of 1780 °C—1 h—30 MPa, during which the flexural strength and fracture toughness reached 556 MPa and 13.5 MPa m^1/2^. Although SiC whiskers exhibited a positive side in terms of toughening Si_3_N_4_ matrix materials [29], its relative density and the grain bonding strength between SiCw and Si_3_N_4_ grains are poor under sintering with low temperature and short holding time. Meanwhile, the application of SiC whiskers not only extra increases the costs and difficulty of Si_3_N_4_ ceramic materials during the preparation process [28,29,30], but also brings damage to health [22].

Additionally, much research [31,32,33] has indicated that the bimodal distribution [34] of Si_3_N_4_ ceramics with more columnar β-Si_3_N_4_ grains is advantageous to improve the strength and toughness by introducing low-cost β-Si_3_N_4_ [35] whiskers [27] and/or seeds [36]. In addition, generally, various reinforced particles such as TiC [37], TiN [5,27], Ti(C, N) [11], WC [38], SiC [4] and graphene [39] are also mixed into Si_3_N_4_-based ceramics [35] to enhance their properties. For example, graphene [21,32,39] was used to reinforce a Si_3_N_4_ ceramics matrix, from which the fracture toughness was markedly enhanced. Si_3_N_4_-TiC ceramic composites [40] were prepared by adding TiC particles and gas-pressure sintering at 1750 °C, which achieved a fracture toughness of 8.4 MPa.m^1/2^. However, compared with synergistic reinforcements [30,41], the individual reinforcing approach is limited in terms of the enhancement of mechanical properties, no longer able to meet the harsh service conditions of Si_3_N_4_ ceramic tool materials [42]. As a result, synergistic reinforcement is an effective method for preparing Si_3_N_4_ ceramic tool materials with outstanding mechanical properties via hot-pressed sintering under low temperature and short holding time.

In the present study, all samples were fabricated by using hot-pressed sintering under low temperature and short holding time (1600 °C—30 min—40 MPa). As-prepared Si_3_N_4_ ceramics were reinforced by introducing β-Si_3_N_4_ whiskers and TiC particles, respectively. As the comparison groups of synergetic-reinforced samples, for the other two samples, we adopted the whisker-reinforced method and particle-reinforced method by adding β-Si_3_N_4_ whiskers and TiC particles, respectively. Subsequently, the effect of the α-β phase transformations, microstructural changes, added β-Si_3_N_4_ whiskers and TiC particles on the Si_3_N_4_ ceramics were detailed examined according to the phase composition, microstructure, mechanical properties and crack propagation paths.

## 2. Experimental Procedure

**Figure 1:**
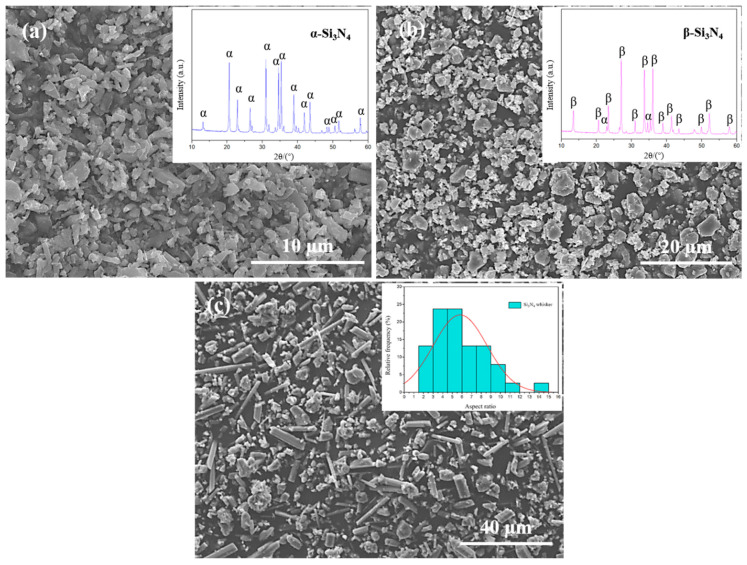
Morphology images of α-Si_3_N_4_ powder (**a**), β-Si_3_N_4_ equiaxed seeds (**b**) and β-Si_3_N_4_ whiskers (**c**) used in experiment, and the corresponding XRD patterns and aspect ratio histogram of β-Si_3_N_4_ whisker (the red curve is the Gaussian fitting curve exhibited the distribution situation of β-Si_3_N_4_ whisker aspect ratio) inserted in the upper right.

The initial materials were commercially available α-Si_3_N_4_ powder (purity ≥ 93%, D_50_ = 1.5 μm, Oxygen content ≤ 1.5%) and β-Si_3_N_4_ equiaxed seeds (purity > 99%) purchased from the Yantai Tomley Hi-Tech Advanced Materials Co., Ltd., Yantai, China. β-Si_3_N_4_ whiskers (purity > 85%, diameter: 1~3 um, length: 5~20 um, Xingtai Nangong New Materials, Ltd., Nangong, China), AlN (purity > 99.99%), ZrN (purity > 99.50%), Y_2_O_3_ (purity > 99.99%), Al_2_O_3_ (purity > 99.99%), and TiC (purity > 99.50%, Qinhuangdao Eno High-tech Materials Development Co., Ltd., Qinhuangdao, China). The SEM morphology images of β-Si_3_N_4_ seeds and whiskers are shown in Figure 1. The D_50_ aspect ratio of β-Si_3_N_4_ whiskers is close to 6:1, which was counted from the corresponding SEM image using Nano Measurer software 1.2.

As listed in Table 1, the content of Y_2_O_3_ as sintering aids is fixed at 5 wt% according to the research on Y_2_O_3_ additive concentration [7]. Based on a previous study [31], 5 wt% AlN-5 wt% ZrN-5 wt% Al_2_O_3_ as a sintering additive was introduced into sample S-2 for further improving its sinterability. The formulas of the samples named S-1, S-2 and S-3 refer to the addition of 5 wt% β-Si_3_N_4_ whiskers, 20 wt% TiC, and 5 wt% β-Si_3_N_4_ seeds-5 wt% TiC-10 wt% β-Si_3_N_4_ whiskers, respectively. In order to obtain a homogeneous slurry, the raw powders and reinforced phases of the three samples were added to nylon cans, after which alumina milling balls and ethyl alcohol solvent were poured into the mixtures of raw powders. Next, the mixtures of raw powders were continuously ball-milled for 8 h at a rotation rate of 300 r/min by using a planetary ball mill. After ball-milling, the acquired slurries were dried by keeping them in a drying oven for 24 h. Subsequently, the dried mixture powders were sieved through a 180-mesh sieve. With regard to the sintering step, the mixed homogeneous ingredients were filled into a graphite mold and sintered by hot-pressing in a graphite resistance furnace (ZT-60-23Y, Chenhua Technology Co., Ltd., Shanghai, China) under vacuum conditions. The heating rate was 15 °C/min before 1200 °C, 10 °C/min at 1200 °C~1500 °C and 5 °C/min at 1500 °C~1600 °C, respectively. The holding-temperature stage was conducted at 1600 °C for 30 min with a uniaxial pressure of 40 MPa. The cooling process of specimens was conducted within the furnace by naturally cooling. Then the as-sintered Si_3_N_4_ ceramic bulks were treated by cutting, grinding and polishing on a buffing machine for the testing trial step.

The practical density was measured using Archimedes’ method, and the relative density was calculated from the ratio of theoretical density to measured density. An X-ray diffractometer (XRD, Rigaku Ultima IV, Japan) was used for phase identification using Cu Kα radiation (λ = 1.54 Å) and a scanning speed of 10°/min with a voltage of 40 kV and current of 40 mA. The weight fraction of the β-Si_3_N_4_ phase was calculated based on the diffraction peak intensity ratios of β/(α + β) proposed by Gazzara and Messier [4]. The consolidated samples were cut into rectangular bar shapes with approximate sizes of 2 × 3 × 20 mm by using an inside diameter slicer (J5060-F, Shanghai Huisheng Electronic Machinery Equipment Co., Ltd., Shanghai, China). Every surface of the test specimens was ground and polished into a mirror surface. Based on the three-point bending method, the flexural strength of each sample was measured from more than five test bars by using a mechanical property testing machine (CDW-5, 5 KN, Changchun Chaoyang Test Instrument Co., Ltd., China) for fine ceramics with a loading speed of 0.5 mm/min and span length of 10 mm. The Vickers hardness and fracture toughness (KIC) were tested using the indentation method with indentation testing equipment (MH-6, Everone Co., Ltd., Shanghai, China) with a load of 98 N and a dwell time of 15 s. The length of the indentation cracks was measured under a scanning electron microscope. Thus, according to the length of the cracks generated by the Vickers indention, the KIC was calculated using an equation proposed by Evan and Charles [25,43]. The final results of Vickers hardness and fracture toughness were calculated from the mean value of at least five indentations at different locations. The plasma-etching process was conducted on the samples in CF_4_/O_2_ gases. The microstructure of the samples was characterized via field emission scanning electron microscopy (SEM, Regulus8100, Leo Company, Germany) under an accelerating voltage of 15 kV.

## 3. Results and Discussion

### 3.1. Phase Composition and Microstructure

**Figure 2:**
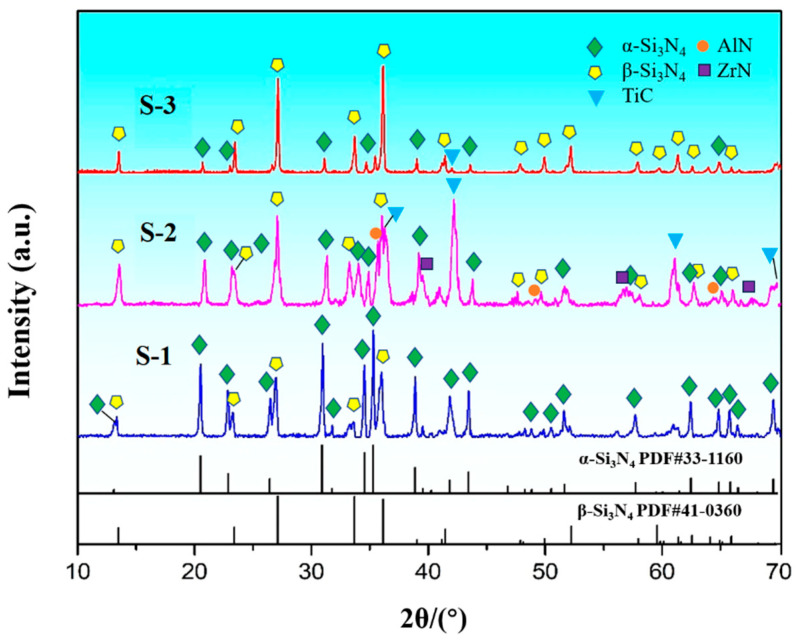
XRD patterns of the compact samples S-1, S-2 and S-3.

The XRD patterns of the sintered bulk samples are presented in Figure 2. The PDF standard cards of the α- and β-Si_3_N_4_ phases are plotted at the bottom of the XRD graph. By analyzing the XRD results of three samples, β-Si_3_N_4_ phases were detected in all samples. The existing α-Si_3_N_4_ phase implies the incomplete phase transformations of the Si_3_N_4_ ceramics. Furthermore, the introduced TiC reinforced phases were also detected in S-2 and S-3 samples apart from the α-Si_3_N_4_ and β-Si_3_N_4_ phases. Based on the detected XRD results of sample S-2, the added ZrN and AlN phases were found.

Based on the diffraction peak intensity of the detected XRD patterns, the α-Si_3_N_4_ and β-Si_3_N_4_ phase contents were estimated by calculating the diffraction peak intensity from the sintered Si_3_N_4_ ceramics at the (102) and (210) planes of the α-phase and the (101) and (210) planes of β-phase [23]. Subsequently, the computed α-/β-Si_3_N_4_ phase contents and relative density of each sintered sample are listed in Table 2. These results indicate that the mass fractions of the β-phase gradually increased from sample S-1 to S-3, while the α-phase content shows an opposite trend. Sample S-3 has a maximum β-Si_3_N_4_ phase content of 79.52% according to the results of β-phase content, resulting from the introduced 5 wt% β-Si_3_N_4_ seeds, which promoted the phase transformation of α- to β-Si_3_N_4_. Consequently, sample S-3 presents a maximum relative density of 97.15% when comparing the relative density values of the three samples in Table 2.

**Figure 3:**
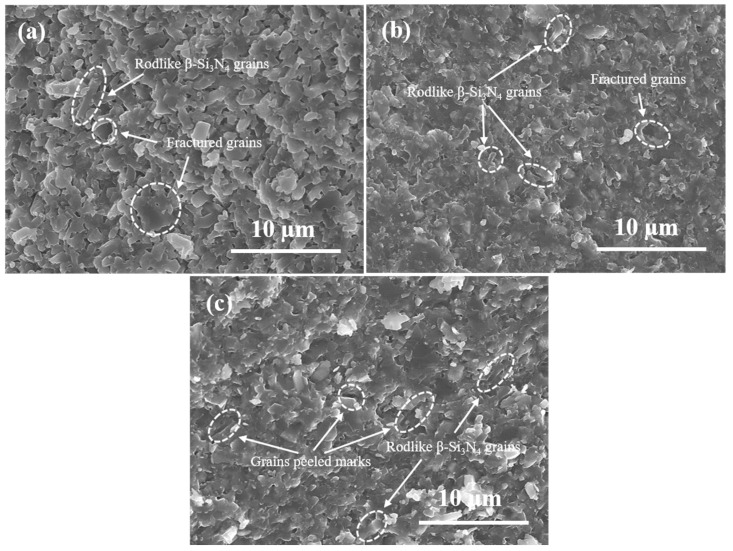
Microstructure pictures of fracture surfaces of the samples S-1 (**a**), S-2 (**b**) and S-3 (**c**). (The white dashed line labels the fractured modes of β-Si_3_N_4_ grains and β-Si_3_N_4_ whiskers in Si_3_N_4_ matrix).

Figure 3 displays the microstructure of the fracture surface of three samples taken from their fractured strip. These three fracture pictures present different microstructural distributions and fracture appearances due to their different raw contents. The microstructure of sample S-1 with 5 wt% β-Si_3_N_4_ whiskers, as shown in Figure 3a, is not dense enough because the coarse β-Si_3_N_4_ whiskers affected the dissolution and precipitation process of the Si_3_N_4_ powder, and the resultant loose microstructure caused the low mechanical properties. Figure 3b shows the microstructure of sample S-2 with added 20 wt% TiC particles and a binary nitride system, which generated many finer equiaxed particles owing to the restriction of more of the TiC particles to Si_3_N_4_ grains. Moreover, the binary nitride additives added into sample S-2 improved its sintering and phase transformation, while the strength and toughness of Si_3_N_4_ ceramics are low. Nevertheless, the microstructure of sample S-3, as shown in Figure 3c, exhibited bigger and longer grains as a result of the addition of β-Si_3_N_4_ seeds and whiskers. Compared with sample S-1, the added β-Si_3_N_4_ seeds can further promote the phase transformation and grain growth of β-Si_3_N_4_ in sample S-3. As a result, the fracture microstructure of sample S-3 with β-Si_3_N_4_ seeds, β-Si_3_N_4_ whiskers and TiC particles presented more fracture modes resulting from the rodlike β-Si_3_N_4_ grains and β-Si_3_N_4_ whiskers than samples S-1 and S-2, which are indicated by white dashed lines.

### 3.2. Grain and Element Distributions of Sintered Samples

**Figure 4:**
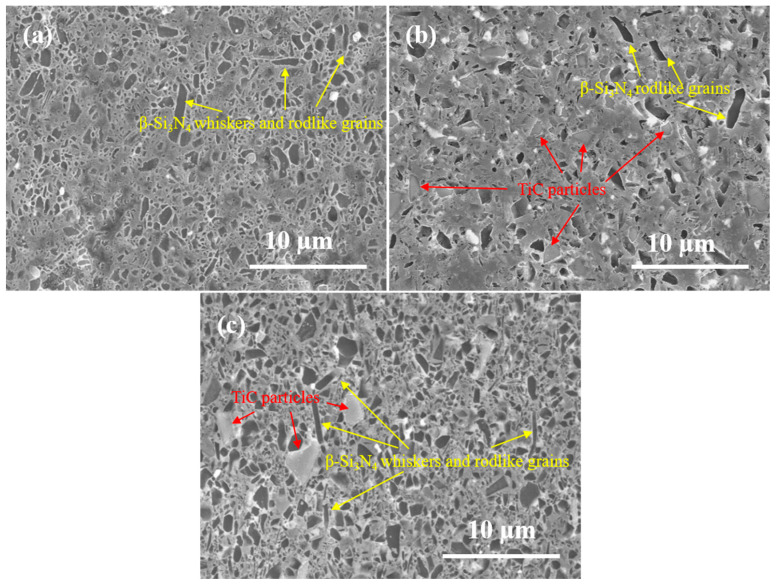
Polished and plasma-etched surfaces of samples S-1 (**a**), S-2 (**b**) and S-3 (**c**) and red and yellow indicated the locations of TiC particles, β-Si_3_N_4_ whiskers and rodlike grains.

For observing the distribution of the Si_3_N_4_ grains, the polished surfaces of as-sintered samples were plasma-etched in CF_4_/O_2_ gases. The SEM images of the plasma-etched surfaces are shown in Figure 4. The left dark areas in the figure are the traces of etching, revealing the outline and position of Si_3_N_4_ grains, and the etched β-Si_3_N_4_ whiskers are indicated by yellow arrows. The unetched TiC particles are marked by red arrows. As seen from Figure 4a, only a few β-Si_3_N_4_ whiskers emerged in sample S-1 with 5 wt% β-Si_3_N_4_ whiskers, while most Si_3_N_4_ grains included α- and β-Si_3_N_4_ as their equiaxed structures. Figure 4b shows the grain composition and distribution of sample S-2 with 20 wt% TiC particles. The etched areas were the fewest owing to the addition of the 20 wt% TiC particles. The black holes with irregularly shaped reveal the locations of the etched Si_3_N_4_ grains. Additionally, the elongated Si_3_N_4_ grains were too few to form a self-reinforced structure, resulting in the poor mechanical properties of sample S-2. As for sample S-3 in Figure 4c, some finer columnar β-Si_3_N_4_ grains are distributed, formed under the hot pressure of 40 MPa and a few elongated β-Si_3_N_4_ grains in the matrix. Apparently, the morphology microstructure of sample S-3 with fine and elongated β-Si_3_N_4_ grains produced a remarkable bimodal microstructure distribution that can improve the mechanical properties of Si_3_N_4_ ceramic materials.

**Figure 5:**
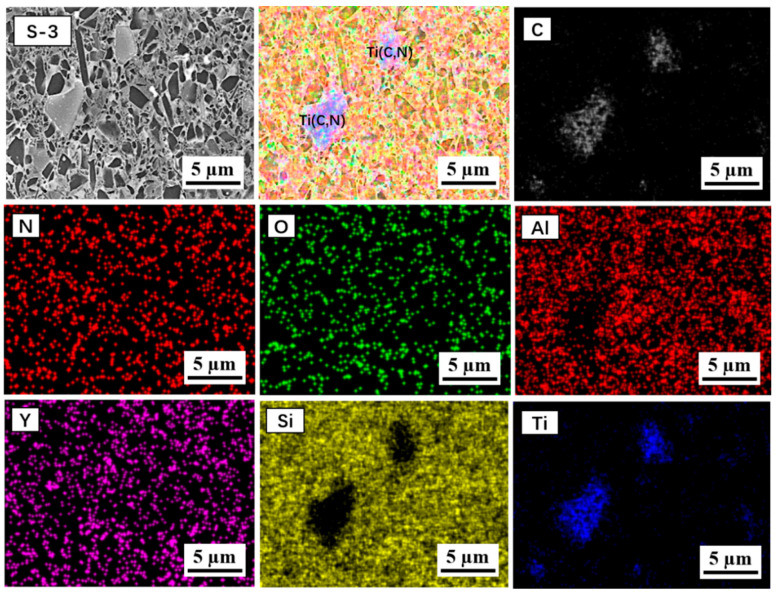
EDS elemental mapping of etched sample S-3 surface, showing the distribution of elemental composition in microstructure.

To further present the distribution situation of elements and grains of the sample S-3, EDS elemental mapping of the etched surface was carried out. The SEM photo of the etched surface and corresponding identified elemental photos (C, N, O, Al, Y, Si, Ti) are shown in Figure 5. The in-added TiC particles with large sizes are distinguished clearly in terms of the mapping distribution of the Ti and C elements, which separately embedded in the Si_3_N_4_ matrix. Furthermore, the distribution of O, Al and Y elements reveals that the additives as boundary phases were evenly dispersed among the Si_3_N_4_ grains.

### 3.3. Vickers Indentation and Crack Propagation Analysis

**Figure 6:**
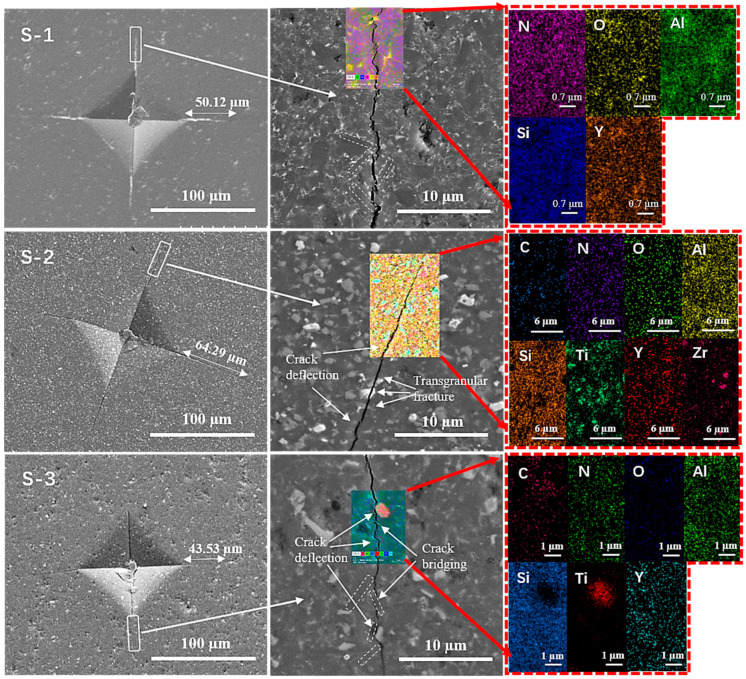
SEM images of Vickers indentation and magnified crack propagation paths, and corresponding EDS mapping photos detected from the partial crack of three samples (S-1, S-2 and S-3).

To assess the influence of synergistic reinforcement on Vickers indentation and crack propagation of the Si_3_N_4_ matrix, the indentation microstructure, crack propagation and elemental mapping in the crack pathway were characterized. Figure 6 exhibits the Vickers indentation, crack propagation and corresponding EDS mapping of the three samples. The indentation with a regular diamond shape is clearly displayed in the image of every sample. The indentation and crack length of samples S-1, S-2 and S-3 can be measured by the indentations in the SEM pictures, which are 50.12, 64.29 and 43.53 μm in length, respectively. One of four cracks in the Vickers indentations was magnified to analyze the strengthening and toughening mechanism. As displayed in the magnified images, some rod-like β-Si_3_N_4_ grains and whiskers in the crack routes are marked by white dotted lines. Moreover, these white arrows point out the positions of toughness behaviors, from which emerged different fracture modes such as crack deflection, crack bridging, trans-granular fractures, etc. Sample S-3 showed the shortest crack length of 43.53 μm when comparing the crack length and route of the three samples, which implies that the crack spreading was suppressed by the larger energy consumption of the reinforced phases. Sample S-2 produced the longest and straightest crack with a crack length of 64.29 μm resulting from the less elongated β-Si_3_N_4_ grains. The crack length of Sample S-1 is between that of samples S-2 and S-3, which contributes to the introduction of β-Si_3_N_4_ whiskers. Additionally, based on the results of EDS element mapping located in the partial cracks, the crack propagation of Sample S-1 mainly follows the grain boundary according to the collected elemental composition of the sintering aids (O, Al, Y elements). Compared the crack SEM images of the three samples, it is obviously seen that the crack propagation of sample S-2 is straighter than that of samples S-1 and S-3. Moreover, eight elements were found from the sintering aids (O, N, Al, Y elements), reinforced particles (C, N, Ti, Zr elements) and Si_3_N_4_ matrix (N, Si elements) of sample S-2. According to the magnified crack and element mapping images of sample S-3, the cracks of sample S-3 exhibit a more winding path resulting from more obstacles such as the TiC particles, elongated β-Si_3_N_4_ grains and whiskers. Therefore, many reinforcing mechanisms occurred in the crack spreading of sample S-3, including crack bridging, crack deflection, trans-granular fracture, whiskers pullout and fracture bifurcation, which contributed to consume more crack propagation energy and enhance the fracture toughness. As a result, sample S-3 presents the optimum mechanical properties.

**Figure 7:**
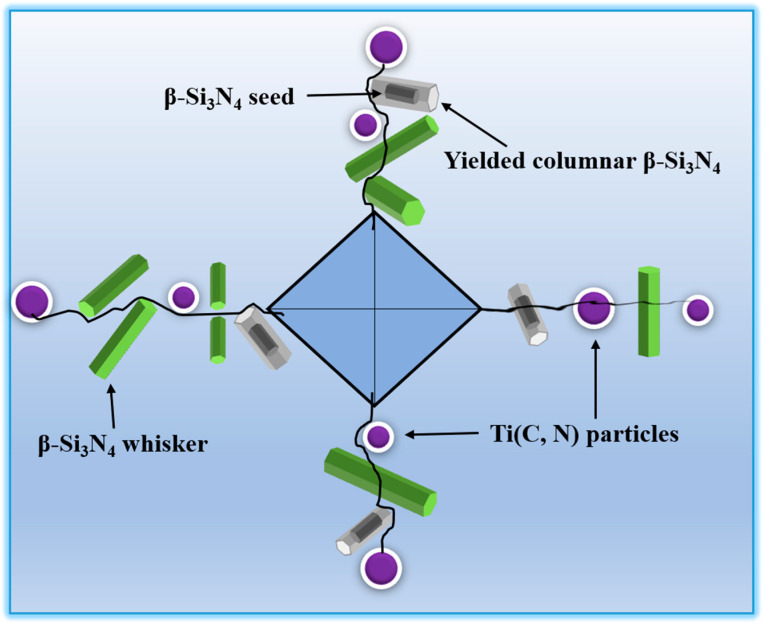
Schematic of the crack propagation induced by indentation.

In order to further exhibit the mechanism of collaborative reinforcement, Figure 7 is a diagram of the indentation and crack propagation of sample S-3 with β-Si_3_N_4_ seeds, β-Si_3_N_4_ whiskers and TiC particles. In the Si_3_N_4_ matrix of sample S-3, there are three kinds of reinforced phases: β-Si_3_N_4_ whiskers, elongated β-Si_3_N_4_ grains and TiC particles. These reinforced phases effectively suppressed crack spreading by consuming considerable fracture energy while generating various strengthening and toughening modes such as trans-crystalline fracture, crack deflection, crack bridging and whiskers extraction, etc. Notably, the residual stress caused by the TiC grains also contributed to the enhancement of mechanical properties. The few elongated β-Si_3_N_4_ grains were formed from the added β-Si_3_N_4_ seeds, which led to the formation of Si_3_N_4_ bimodal structure. Consequently, the synergistic toughening of Si_3_N_4_ ceramic tool materials with low-temperature sintering was realized by introducing β-Si_3_N_4_ whiskers and TiC particles as composite reinforced phases.

### 3.4. Mechanical Properties

**Figure 8:**
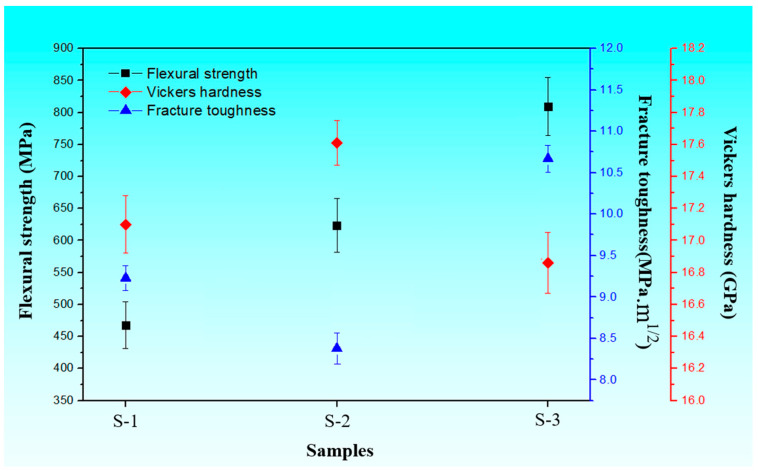
Line chart of flexural strength, fracture toughness and Vickers hardness of samples S-1, S-2 and S-3.

Figure 8 shows a line chart of the mechanical properties of the three sintered samples. The change trends in flexural strength, Vickers hardness and fracture toughness of three samples are simply observable. The flexural strength of sintered samples exhibits an increased tendency from sample S-1 to S-3. In addition, sample S-1 with added 5 wt% Si_3_N_4_ whiskers exhibits ordinary mechanical properties, namely a flexural strength of 623.69 ± 42.06 MPa, Vickers hardness of 17.30 ± 0.18 GPa and fracture toughness of 9.23 ± 0.15 MPa.m^1/2^. The compact sample S-2 with added 10 wt% AlN/ZrN-20 wt% TiC presents the lowest fracture toughness, improved flexural strength and the maximum Vickers hardness of 17.61 ± 0.14 GPa. In comparison, sample S-3 with added multiple reinforced phases displays the highest flexural strength of 809.10 ± 45.59 MPa, fracture toughness of 10.67 ± 0.16 MPa.m^1/2^ and Vickers hardness of 16.86 ± 0.19 GPa, which resulted from the collaborative reinforcement of β-Si_3_N_4_ seeds, β-Si_3_N_4_ whiskers and TiC particles.

Based on the obtainable results from our references, the mechanical properties data of Si_3_N_4_-based ceramics with various additives prepared using different sintering conditions and technology are summarized and presented in Table 3. According to the statistical results, the as-sintered samples of this study presented excellent comprehensive mechanical properties. Additionally, in contrast, the sintering conditions with low temperature and short holding time were demonstrated to be effective via synergistic reinforcement for preparing advanced Si_3_N_4_ ceramics. In general, it can be concluded that the simultaneous addition of β-Si_3_N_4_ seeds, β-Si_3_N_4_ whiskers and TiC particles is able to significantly enhance the mechanical properties of Si_3_N_4_ ceramics under hot-pressed sintering with low costs. Meanwhile, it turned out that the efficiency of synergistic reinforcement surpasses that of a single reinforcement.

## 4. Conclusions

Si_3_N_4_ ceramic tool materials with added β-Si_3_N_4_ seeds, β-Si_3_N_4_ whiskers and TiC particles were obtained via hot-pressing sintering at 1600 °C—30 min—40 MP and exhibited excellent comprehensive mechanical properties. Furthermore, the mechanisms of synergistic reinforcement were investigated by analyzing phase compositions, microstructure, crack propagation and reinforcing behaviors. Additionally, the β-Si_3_N_4_ seeds mixed into the Si_3_N_4_-based ceramics effectively boosted the phase conversion of α-Si_3_N_4_ to β-Si_3_N_4_ in the Si_3_N_4_ ceramics. Meanwhile, the elongated β-Si_3_N_4_ grains, β-Si_3_N_4_ whiskers and added TiC particles in the sintered Si_3_N_4_ ceramic matrix were contribute to the improvement of mechanical properties. In terms of crack prolongation, some strengthening and toughening phenomena such as crack bridging, crack deflection, trans-granular fracture, whisker pullout and fracture bifurcation were observed and analyzed. These crack resistance behaviors consumed abundant crack propagation energy. As a result, the synergistic reinforcement applied to the as-prepared Si_3_N_4_ ceramics achieved a synergistic enhancement of flexural strength and fracture toughness (809.10 ± 45.59 MPa and 10.67 ± 0.16 MPa.m^1/2^) while maintaining a fine Vickers hardness (16.86 ± 0.19 GPa). Thereby, the synergistic reinforcement method of Si_3_N_4_ ceramics by adding β-Si_3_N_4_ whiskers and TiC particles effectively toughened the Si_3_N_4_ ceramic materials. The conclusions of this work provide a manufacturing method for Si_3_N_4_ ceramic materials with excellent mechanical properties under hot-pressed sintering with low temperature and short holding time.

## Figures and Tables

**Table 1:** Raw material composition of samples.

Samples	α-Si_3_N_4_ (wt%)	β-Si_3_N_4_ (wt%)	Al_2_O_3_ (wt%)	Y_2_O_3_ (wt%)	AlN (wt%)	ZrN (wt%)	TiC (wt%)	β-Si_3_N_4_w (wt%)
S-1	85	/	5	5	/	/	/	5
S-2	60	/	5	5	5	5	20	/
S-3	70	5	5	5	/	/	5	10

**Table 2:** α/β-Si_3_N_4_ phase contents and relative density of sintered samples.

Samples	α-Phase (wt%)	β-Phase (wt%)	Relative Density (%)
S-1	64.95	35.55	94.92
S-2	50.23	49.27	96.56
S-3	20.48	79.52	97.15

**Table 3:** Comparison of mechanical properties of Si_3_N_4_ ceramics with various additives sintered at different conditions.

Reinforced Phases Composition in Si_3_N_4_ Matrix	Different Sintering Technology	Flexural Strength (MPa)	Fracture Toughness (MPa·m^1/2^)	Vickers Hardness (GPa)	Ref.
5 wt% β-Si_3_N_4_ /10 wt% Si_3_N_4_w/5 wt% TiC	HPS: 1600 °C—30 min—40 MPa	809.10 ± 45.59,	10.67 ± 0.16	16.86 ± 0.19	This study
3 wt% β-Si_3_N_4_ seeds	HPS: 1800 °C—2 h—30 MPa	/	9.7 ± 0.67	/	[36]
90 wt% β-Si_3_N_4_ powder	GPS: 1600 °C—8 h—0.1 MPa	553 ± 22	3.5	/	[35]
30 wt% TiN	SPS: 1600 °C—10 min—20 MPa; HPS: 1700 °C—1 h—20 MPa	/	7.8; 6.8	15.3; 15.1	[5]
5 wt% TiC	GPS: 1750 °C—1 h—2 MPa	436	8.4	17.3	[40]
10 vol.% Ti(C, N); 20 vol.% Ti(C, N)	HPS: 1750 °C—1 h—35 MPa	860 ± 90; 695 ± 60	8.19 ± 0.91; 6.44 ± 0.32	16.29 ± 0.23; 15.84 ± 0.97	[15]
5 vol.% n-TiN/20 vol.% n-Si_3_N_4_w	HPS: 1650 °C—40 min—30 MPa	980	9.6	18	[27]

HPS: hot-pressed sintering; GPS: gas pressure sintering; SPS: spark plasma sintering.

## Data Availability

No new data were created.
